# Cephalometric Soft Tissue Characteristics of Unilateral Cleft Lip and Palate Patients in Relation to Missing Teeth

**DOI:** 10.1155/2017/2392808

**Published:** 2017-10-23

**Authors:** Khalid A. Almoammar, Hala A. Almarhoon, Waeil Batwa, Nasser Alqahtani, Thikriat Al-Jewair, Sahar Albarakati

**Affiliations:** ^1^Department of Pediatric Dentistry and Orthodontics, College of Dentistry, King Saud University, Riyadh, Saudi Arabia; ^2^Department of Orthodontics, Faculty of Dentistry, King Abdulaziz University, Jeddah, Saudi Arabia; ^3^Department of Orthodontics, State University of New York at Buffalo, Buffalo, NY, USA

## Abstract

**Objective:**

This study aimed to evaluate cephalometric soft tissue characteristics in individuals with unilateral complete cleft lip and palate (UCCLP) both with and without missing teeth.

**Design:**

A retrospective investigation of patient records, who are being treated at the cleft lip and palate (CLP) clinics at the College of Dentistry. Ninety-six consecutive records of nonsyndromic UCCLP subjects were recruited (33 subjects without missing teeth and 63 subjects with missing teeth). Linear and angular soft tissue measurements obtained from lateral cephalometric radiographs were evaluated and compared among the studied samples.

**Results:**

Lower lip was significantly retruded and shorter (*p* = 0.037), *p* = 0.015, respectively; in addition to the fact that shallower mentolabial sulcus (*p* = 0.05) was found in the subjects with missing teeth, the rest of the soft tissue was not significantly different between the two groups.

**Conclusion:**

In subjects with a UCCLP anomaly, missing teeth have an effect on lower lip position and length, which influenced the mentolabial sulcus. Lower lip position and length differ between cleft patients who present with either multiple missing teeth or with no missing teeth, and this needs to be considered during orthodontic treatment planning and surgical management for the cleft defect.

## 1. Introduction

Orofacial clefts are considered to be the most common craniofacial anomaly, worldwide [1]. Evidence-based research and anecdotal clinical observations have reported specific adverse effects on craniofacial growth as a result of orofacial anomalies. These are caused by the intrinsic effects of the cleft anomaly and possibly the treatment effects, including the effects of scarring following the surgical closure of the cleft defects [2, 3]. Lisson et al. (1999) believed that these surgical interventions influence the development of the dentition and restrict craniofacial growth, especially skeletally in the anteroposterior dimension [3]. Missing teeth have been reported in individuals with cleft lip and palate (CLP) [4–11]. However, this type of dental anomaly varies according to ethnicity, cleft type, and gender [12–16]. Missing teeth have been linked to various craniofacial consequences, including a class III pattern, maxillary and mandibular angular prognathism, and maxillary restriction [17].

A recent study attempted to explore the association between missing teeth and the skeletal and dental characteristics of CLP subjects [18]. This study evaluated a number of cephalometric characteristics in Taiwanese individuals with unilateral CLP; it found a general reduction in the skeletal vertical dimension and a reduction in the overjet [18].

However, in the literature, no previous investigation has evaluated the effects of missing teeth on soft tissue characteristics among subjects with a UCCLP anomaly. Hence, this study aimed to evaluate cephalometric soft tissue characteristics in individuals with UCCLP, with and without missing teeth.

## 2. Methods

### 2.1. The Study Design

This retrospective study was based on the records of patients attending the Cleft Lip and Palate Clinics, College of Dentistry. Linear and angular measurements obtained from lateral cephalometric radiographs were evaluated and compared among the studied samples. Soft tissue characteristics were assessed in individuals with UCCLP, with missing teeth, and compared to age and gender-matched groups of UCCLP patients without missing teeth.

### 2.2. The Sample

The sample consisted of two groups:

- Group 1: thirty-three UCCLP individuals without missing teeth
- Group 2: sixty-three UCCLP individuals with missing teeth

The patient records were retrieved from the database of the Cleft Lip and Palate Clinic and the orthodontic clinics of the College of Dentistry, between January 1991 and December 2014.

The following inclusion criteria were applied:

1. Individuals with UCCLP, ranging in age from 7 to 14
2. Individuals with complete records, including dental/medical files, panoramic radiographs (orthopantomograms), occlusal radiographs, and lateral cephalometric radiographs

The following exclusion criteria were used:

1. Patients that had undergone comprehensive orthodontic or orthopedic treatment
2. Patients that had undergone any extraction treatment
3. Patients with poor-quality pretreatment records
4. Patients who had bone graft treatment

All patients with CLP were treated by multiple surgeons based on the standard protocol of the Cleft Lip and Palate Team, College of Dentistry.

### 2.3. Methods

Panoramic and occlusal radiographs, which were taken when the patients were 7 to 12 years of age, were interpreted and used to determine if permanent teeth were missing. For standardization purposes, all the selected cephalometric radiographs were taken with the patient's head in a natural position and with the teeth in centric occlusion. The radiographs were taken with a Planmeca Proline XC PAN/CEPH X-Ray Unit (Planmeca Oy, Helsinki, Finland), set at 66–72 kV, 12 mA, and 0.3–1 seconds of exposure time. The magnification ratio at the midsagittal plane was 10.74% in the cephalometric film. The tracing and analysis were performed with indirect digitization using Dolphin Imaging Software® (Version 11.7.05.66, Dolphin Imaging & Management Solutions, Chatsworth, CA, USA). One operator identified the anatomic hard and soft tissue landmarks, and software was used to calculate the linear and angular measurements. The scanning and digitization of the hard copy lateral cephalometric films were done on the university campus using EPSON Perfection V700 Photo, a dual lens system (Epson Electronics Company, Suwa, Japan).

For intraexaminer reliability, 10 randomly selected lateral cephalometric radiographs were traced and measured on two occasions within a two-week interval. Ethical approval was sought and granted from the College of Dentistry Research Center Ethical Committee (number PR 0026), informed consent was obtained.

### 2.4. Cephalometric Landmarks and Measurements

**Figure 1:**
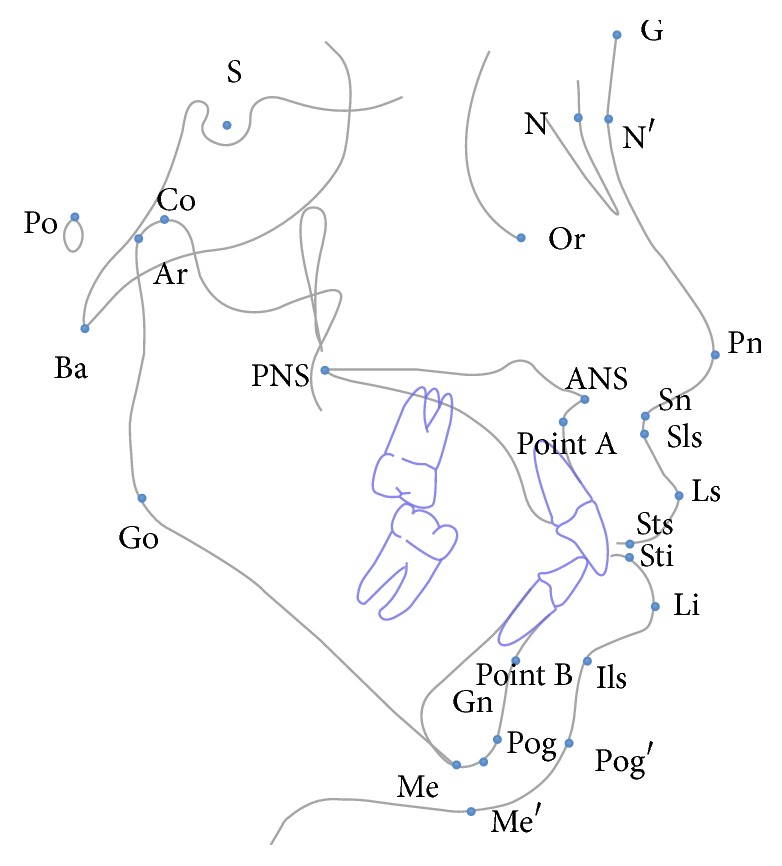
Soft tissue landmarks used in the study; G, soft tissue glabella, the most prominent or anterior point in the midsagittal plane of the forehead at the level of the superior orbital ridges; Sn, subnasale, a point at the junction between the lower border of the nose and the beginning of the upper lip at the midsagittal plane; Pog′, soft-tissue pogonion, the most prominent or anterior point on the soft tissue chin in the midsagittal plane; Me′, soft tissue menton, the inferior soft tissue contour of the chin; N′, soft tissue nasion, the point of the deepest concavity of the soft tissue contour of the root of the nose; A′, soft tissue A-point, the most posterior (deepest) point on the curve of the soft tissue; B′, soft tissue B-point, the deepest point on the bony curvature along the anterior border of the soft tissue chin; Sti, stomion inferius, the most superior point of the lower lip; Sts, stomion superius, the lowest point of the upper lip; Pn, pronasale, the most prominent and anterior point of the nose; Li, labiale inferius, the median point in the lower margin of the lower membranous lip; Ls, labrale superius, the median point in the upper margin of the upper membranous lip.

The selected landmarks are shown in Figure 1. The 18 soft tissue linear and angular measurements are listed and defined in Table 1.

### 2.5. Statistical Analysis

Data were analyzed using the Statistical Package for the Social Sciences (SPSS Version 22.0 for Windows; IBM Corporation, Armonk, NY, USA). Descriptive and analytical statistics were undertaken with the help of a biostatistician. The error test of Dahlberg's formula was used for the reliability analysis. Student* t*-tests were performed to compare the soft tissue measurements between the two groups. A *p* value of, or below, 0.05 was considered to be statistically significant.

## 3. Results

A total of 96 patient records met the study's inclusion criteria and contained the required dental/medical files, panoramic radiographs (orthopantomograms), occlusal radiographs, and lateral cephalometric radiographs. The reliability tests revealed that all the variables were reliable; the *p* value of the difference between the two sets of cephalometric measurements was not significant. The Pearson's correlation values ranged between 0.749 and 0.993, which is considered to be highly reliable. The reliability of repeated measurements (10 samples) of *x* and *y* landmark coordinates which were collected at two different time points was assessed by calculating the margin of error using Dahlberg's formula. The analysis shows “the quantity of error was small enough” for all the variables, which indicates the good reliability of measurements.

This retrospective study used the records of 96 individuals with UCCLP, among whom 44 were male (45.9%) and 52 were female (54.1%) (Table 2). They ranged in age from 7 to 12, with a mean age of 10.94 years.

The reported missing teeth in this study were mainly the lateral incisors on the cleft side followed by premolars and central incisors. The total number of missing teeth in the sample was 79 in 63 patients, including 61 lateral incisors, 14 premolars, and 4 central incisors. The mean number of teeth missing in group 2 is 1.25 per subject. A detailed distribution of the missing teeth is illustrated in Table 3.

No significant difference between the groups was found when evaluating the upper lip. While the lower lips were more retrusive in relation to the S-line among the experimental group than the control group (*p* = 0.037). In addition, the mentolabial sulcus was significantly deeper in controls (*p* = 0.05) and the length of the lower lip was decreased in the experimental group in comparison with the control group (*p* = 0.015). Other variables were also different between the experimental and control groups; however, these variations were not significant. Full details of the soft tissue measurements are presented in Table 4.

## 4. Discussion

Ninety-six consecutive records of individuals with UCCLP attending the Cleft Lip and Palate Team, College of Dentistry, were recruited in this retrospective investigation. No previously published articles have discussed soft tissue variables in CL/P subjects with missing teeth; hence, we aimed to explore this unknown aspect. Our study mainly measured the soft tissue variables of the UCLP with and with no missing teeth. A total of 18 variables were measured in each sample, with 15 in the anteroposterior dimension and three in the vertical dimension, 11 millimetric and seven angular measurements. These measurements evaluated the soft tissue profile and the features of the nose, lips, and chin.

In our study, only UCLP subjects who had not yet undergone bone grafts were included. As bone graft will most certainly influence the skeletal and dental measurements of the patient. According to Chang et al., the SNB, SN-Pog, ANB, lower incisors to mandibular plane, gonial angle, ANS-PNS distance, and A-PNS distance were significantly different between the grafted group and their matching controls [19]. For this reason, our sample excluded UCLP subjects with a history of bone graft operations.

At the age of 7–14 years, all the permanent tooth buds (except for third molars) should be present and the investigation of most anomalies can be easily identified. According to Borodkin et al., CLP patients exhibit a delay in tooth development of 6 months [20] and also De Carvalho Carrara et al. determined a higher mean age for the eruption of lateral incisors and bicuspids [21].

The consistency between the two sets of soft tissue measurements of all 18 variables was measured, and the correlation values ranged from 0.795 to 0.993, which indicates excellent association between the two sets of measurements. Moreover, calculating the margin of errors using Dahlberg's formula showed that the quantity of errors was small enough. All these reflect a highly reliable measuring techniques. Yet some landmarks were not easy to identify primarily because of other structural superimpositions, such as the Ar, Co, Po, and, more frequently, ANS and point A.

Different linear and angular measurements were chosen to analyze the same structures in this study to validate the assessment of these structures. The upper and lower lips positions were measured in relation to S-line and SnV line. In addition, millimetric measurements in both A-P and vertical dimensions verify any faults in the position or the size of the nose, lips, or chin.

In our study, we have chosen the commonly used landmarks with distances and angles to describe the soft tissue profile of any given sample. Three main lines are usually used to assess the lips, E-line, S-line, and subnasale vertical line (SnV). The latter is the most reliable, and it depends only on identifying the true vertical line and the subnasale landmark while tracing the lateral cephalometric radiograph. The former two depend mainly on the position of other structures. The E-line, Ricketts Esthetic plane, depends on the tip of the nose and the soft tissue chin point, which is not very hard to identify. The limitation happens in nonharmonious faces, when the tip of the nose is curved upwards or most probably downwards and/or when the soft tissue chin point is at fault anteroposteriorly, which is common in CL/P subjects. Steiner tried to overcome this limitation by using the S-line, which eliminates the effect of the vertical position of the tip of the nose and some of the horizontal for this reason subnasale vertical line was used in this study and was supplemented by S-line. Same applies to upper lip [22].

In general, Wu et al. concluded that craniofacial morphology was not significantly different among patients without congenitally missing teeth and those with one congenitally missing tooth, although soft tissues variables were not assessed in their study [18]. According to our study, there were no major soft tissue differences between the experimental groups and the control group in the anteroposterior dimension, with the exception of the lower lip retrusion in relation to the S-line. The lower lip attempts to achieve labial seal, and touching the upper lip may explain this because the upper lip is getting more retrusive with the presence of missing teeth, and it is trying to follow its skeletal base (the maxilla), which is retruded in CL/P subjects [23, 24]. The mentolabial sulcus was significantly different among the subjects in our sample; it was shallower in the experimental group, possibly because the lower lip is too stretched as it tries to be as close as possible to the upper lip, which is slightly retrusive according to the S-line. Vertically, the lower lip length decreases in the test group; the decreased bone support and overclosure could be the reasons, which can be confirmed by the decrease in the interlabial gap among the missing teeth group (Table 4), interestingly, upper lip length was decreased as well in group 2, yet this was not significant.

The NLA was not different among our groups; the NLA was not affected when missing teeth complicates the anomaly. According to Brudnicki et al., different surgical techniques to repair the cleft palate are associated with different NLA among specially among preadolescent patients with UCLP; these include vomerplasty and Langenbeck technique [25].

The upper lip position was retrusive to all reference lines; however, this was not significant between the two groups; same applies to the length of the upper lip. This could be due to the minimal effect that missing teeth have on the profile. The upper lip length in cleft subjects is initially short [26], and missing teeth in the upper arch do not further affect the lip length.

The lower lip was the most affected of all soft tissue structures in both anteroposterior and vertical planes in our study, as mentioned earlier, and its relation with the chin, therefore, was also affected; the mentolabial sulcus was shallower in the group having missing teeth. The chin itself was not affected, though. The chin's position to the SnV line and its thickness were not affected. The skeletal chin point position can be masked by the soft tissue thickness, making the profile of a subject better or worse. Soft tissue profile angles, despite the method of assessment, did not show significant differences between the two groups of our sample. No additional effects on the soft tissue convexity of the UCLP subjects were observed when missing teeth complicate the anomaly. The nasofacial angle, nasomental angle, and nasofrontal angle, although reported in the dental literature, are not widely used. Going back to the literature, many different variables have been used to evaluate the soft tissue covering of the skeletal and dental structures. Many of these variables, if not all, have a wide range, and although they are all within normal limits, the results vary.

This is a retrospective study, which is one of its limitations; the small sample size is another limitation. This study may have also benefited from frontal and profile photographs, which are valid methods used to evaluate the nose, lips, and chin. Moreover, the use of submental-vertical views to evaluate the nostrils in subjects with cleft defects would be valuable [27]. The initial sample size of our study was not calculated because of the wide range of standard deviations values among the different variables measured. Although the current sample size is relatively low, it has to be considered that this was a single-centered study that looked at a particular type of cleft lip and palate anomaly. Ideally, a multicenter study would be preferred to collect a larger sample size with variable types of cleft to investigate craniofacial anomaly. The relatively low sample size is a consequence of the strict inclusion criteria applied in the current study. The final sample size was 96, which is comparable to previously published data in the dental literature [28, 29].

## 5. Conclusion

CLP subjects with the known soft tissue characteristics of their anomaly may experience additional complications when they have missing teeth in the upper arch. Although the majority of soft tissue variables were not statistically significant between our experimental and control groups of the sample, some were significant, such as the lower lip retrusion, mentolabial sulcus depth, and the length of the lower lip.

## Figures and Tables

**Table 1:** Soft tissue measurements used in the study.

	Variable measured	Definition	Definition using landmarks
*Anteroposterior measurements*			
(1)	Upper lip to S-line (mm)	Position of the upper lip to a line passing through a midpoint between the pronasale and subnasale and soft tissue Pog	Ls to S-line
(2)	Lower lip to S-line (mm)	Position of the lower lip to a line passing through a midpoint between the pronasale and subnasale and soft tissue Pog	Li to S-line
(3)	Upper lip to Sn V (mm)	Position of the upper lip to a line parallel to a true vertical line passing through the subnasale	Ls to SnV line
(4)	Lower lip to Sn V (mm)	Position of the lower lip to a line parallel to a true vertical line passing through the subnasale	Li to SnV line
(5)	Chin to Sn V (mm)	Soft tissue Pog′ to a line parallel to true a vertical line passing through the subnasale	Pog′ to SnV line
(6)	Facial convexity angle°	Convexity of the face (without the nose)	N′ B′ A′
(7)	Angle of facial convexity°	Convexity of the face (with the nose)	Pn A′ Pog′
(8)	Mentolabial sulcus (mm)	The distance from the lower chin lower lip line to the maximum depth of the mentolabial sulcus	Li Pog′ line to the sulcus
(9)	Chin thickness (mm)	The distance between the soft tissue and hard tissue Pog	Pog-Pog′ distance
(10)	Nasofacial angle°	The angle between the glabella-soft tissue nasion and tangent to midnose	G-Pog′ line and midnose tangent
(11)	NLA°	Nasolabial angle	Pn-Sn-Ls
(12)	Nasomental angle°	Soft tissue nasion, the tip of the nose, soft tissue chin	N′ Pn Pog′
(13)	Nasal projection°	Angle formed by the soft tissue nasion, tip of the nose, and subnasale	N′ Pn Sn
(14)	Nasal length (mm)	Distance from the tip of the nose to the subnasale	Pn-Sn distance
(15)	Nasofrontal angle°	The angle between the glabella-soft tissue nasion line and tangent of the midnose	G-N′-midnose tangent

*Vertical measurements*			
(16)	Interlabial gap (mm)	Distance between the upper and lower lips	Sts- Sti distance
(17)	Upper lip length (mm)	Vertical distance of the upper lip	Sn-Sts distance
(18)	Lower lip length (mm)	Vertical distance of the lower lip	Sti Me′ distance

**Table 2:** Descriptive data of the sample.

	Gender		Side of cleft		Total sample	
	Male	Female	Right	Left	Group 1	Group 2
Number	44	52	46	50	33	63
Percentage %	45.9	54.1	47.9	52.1	34.4	65.6
Total number (%)	*96 (100%)*		*96 (100%)*		*33 (34.4%)*	*63 (65.6%)*
					*96 (100%)*	

**Table 3:** The distribution of missing teeth of the sample.

Combination of teeth missing in the sample subjects	Number of subjects	%
Lateral incisor only	44	69.8
Central incisor and lateral incisor	3	4.8
Lateral incisor and premolar	8	12.7
Lateral incisors and premolar	2	3.2
Central incisor	1	1.6
Premolar	4	6.3
Laterals incisors	1	1.6
Total	63	100

**Table 4:** Student *t*-tests were performed to compare the soft tissue measurements between the two groups; lower lip to S-line, mentolabial sulcus depth, and lower lip length values were significantly different between group 1 and group 2.

	Variable	Group 1 Mean ± SD	Group 2 Mean ± SD	*p*-value
*Soft tissue anteroposterior measurements*				
(1)	Upper lip to S-line (mm)	0.32 ± 2.33	−0.31 ± 2.34	0.310
(2)	Lower lip to S-line (mm)	5.21 ± 2.97	3.77 ± 2.68	0.037^*∗*^
(3)	UL to Sn V (mm)	2.45 ± 2.29	1.85 ± 2.62	0.263
(4)	LL to Sn V (mm)	3.19 ± 4.20	2.20 ± 4.70	0.258
(5)	POG to Sn V (mm)	−7.43 ± 5.86	−6.64 ± 6.8	0.832
(6)	Facial convexity angle°	10.81 ± 3.47	11.70 ± 2.97	0.369
(7)	Angle of facial convexity°	148.39 ± 83.94	133.87 ± 103.9	0.737
(8)	Mentolabial sulcus (mm)	16.11 ± 6.58	13.08 ± 5.89	0.050^*∗*^
(9)	Chin thickness (mm)	11.60 ± 3.04	11.04 ± 3.32	0.873
(10)	Nasofacial angle°	29.12 ± 4.06	28.91 ± 4.17	0.608
(11)	NLA°	107.78 ± 12.76	107.3 ± 11.46	0.720
(12)	Nasomental angle°	135.12 ± 6.71	135.41 ± 6.79	0.638
(13)	Nasal projection°	13.12 ± 2.91	13.71 ± 2.62	0.262
(14)	Nasal length (mm)	17.61 ± 2.42	17.84 ± 2.60	0.641
(15)	Nasofrontal angle°	145.78 ± 7.47	144.88 ± 7.75	0.627

*Soft tissue vertical measurements*				
(16)	Interlabial gap (mm)	4.39 ± 3.64	3.53 ± 2.19	0.418
(17)	Upper lip length (mm)	20.16 ± 2.34	19.57 ± 4.37	0.128
(18)	Lower lip length (mm)	46.63 ± 5.86	43.02 ± 7.02	0.015^*∗*^

^*∗*^Statistically significant values.
